# Platelet-Rich Plasma Derived Growth Factors Contribute to Stem Cell Differentiation in Musculoskeletal Regeneration

**DOI:** 10.3389/fchem.2017.00089

**Published:** 2017-10-31

**Authors:** Yun Qian, Qixin Han, Wei Chen, Jialin Song, Xiaotian Zhao, Yuanming Ouyang, Weien Yuan, Cunyi Fan

**Affiliations:** ^1^Department of Orthopedics, Shanghai Jiao Tong University Affiliated Sixth People's Hospital, Shanghai, China; ^2^Shanghai Sixth People's Hospital East Campus, Shanghai University of Medicine and Health, Shanghai, China; ^3^Renji Hospital, School of Medicine, Shanghai Jiao Tong University, Shanghai, China; ^4^School of Pharmacy, Shanghai Jiao Tong University, Shanghai, China

**Keywords:** platelet-rich plasma, growth factors, stem cells, cell differentiation, musculoskeletal regeneration

## Abstract

Stem cell treatment and platelet-rich plasma (PRP) therapy are two significant issues in regenerative medicine. Stem cells such as bone marrow mesenchymal stem cells, adipose-derived stem cells and periodontal ligament stem cells can be successfully applied in the field of tissue regeneration. PRP, a natural product isolated from whole blood, can secrete multiple growth factors (GFs) for regulating physiological activities. These GFs can stimulate proliferation and differentiation of different stem cells in injury models. Therefore, combination of both agents receives wide expectations in regenerative medicine, especially in bone, cartilage and tendon repair. In this review, we thoroughly discussed the interaction and underlying mechanisms of PRP derived GFs with stem cells, and assessed their functions in cell differentiation for musculoskeletal regeneration.

## Introduction

Musculoskeletal regeneration requires growing development of stem cell technology. The supply of necessary cells derived from sterile sources in physiological conditions has provided us with an unlimited and sustainable pattern in the field of cell replacement treatment and other applications (Deshpande et al., 2013). The stem cell therapy contributes to proliferative sustainability, multidirectional differentiation and anoxia endurance (Wang et al., 2016). Different tissues have been used as stem cells sources. Doctors have succeeded in making progress in many cells, such as bone marrow mesenchymal stem cells, adipose-derived stem cells and peripheral blood stem cells (Jeevanantham et al., 2012; Delewi et al., 2013). For instance, adipose-tissue derived stem cells (ADSCs) have vast therapeutic potential. On occasion, they have been applied clinically, for example, in maxillary reconstruction, critical limb ischemia or insulin-dependent diabetes mellitus. Besides, its self-renewing and differentiation in different cellular lines, including tendon and bone tissue is very important in many aspects (Ahmad et al., 2012). *In vivo* studies showed that mesenchymal stem cells (MSCs) can self-renew or differentiate into multiple lineages and regenerate bone, cartilage, muscle, blood, and cardiac tissue (Longo et al., 2013). They found that the transplanted cells improved the function of injured organs by attracting the host's repair cells to the damaged site by releasing certain factors (i.e., VEGF) that indirectly helped in the repair process. However, the exact source of these host repair cells was not fully elucidated. Besides, researchers have concentrated on the part of MSCs as an important trauma-related factor in vasculogenesis and osteogenesis (Gawlitta et al., 2012; Askarinam et al., 2013). In other words, MSC is directed to the damaged site and functions as a regulator for vasculogenesis and osteogenesis (Levi et al., 2011). Moreover, scientists believe that stem cells have huge potential in the course of cell recombination. Human stem cells can proliferate infinitely *in vitro*, thereby providing samples with reproducible characteristics (Thangarajah et al., 2009).

Although the therapeutic effect of stem cell is very promising, its low efficiency has compromised its potential applications in many aspects (Kang et al., 2012). People tried their best to use priming for improving the therapeutic efficacy of stem cell, but failed due to setbacks like low efficient improvement and an immune deficiency of certain proteins. Bone marrow has been identified as an excellent source of MSCs and can be accessed readily by surgeons intraoperatively. Nevertheless, scientists also attempted to obtain MSCs from fatty tissue (Angeline and Rodeo, 2012). During the last decades, scientists used a variety of different vehicles for the local delivery of MSCs, including type I collagen gels, collagen sponges and fibrin, to the Achilles, patellar, or RC tendons in animal models (Majewski et al., 2009).

**Figure 1:**
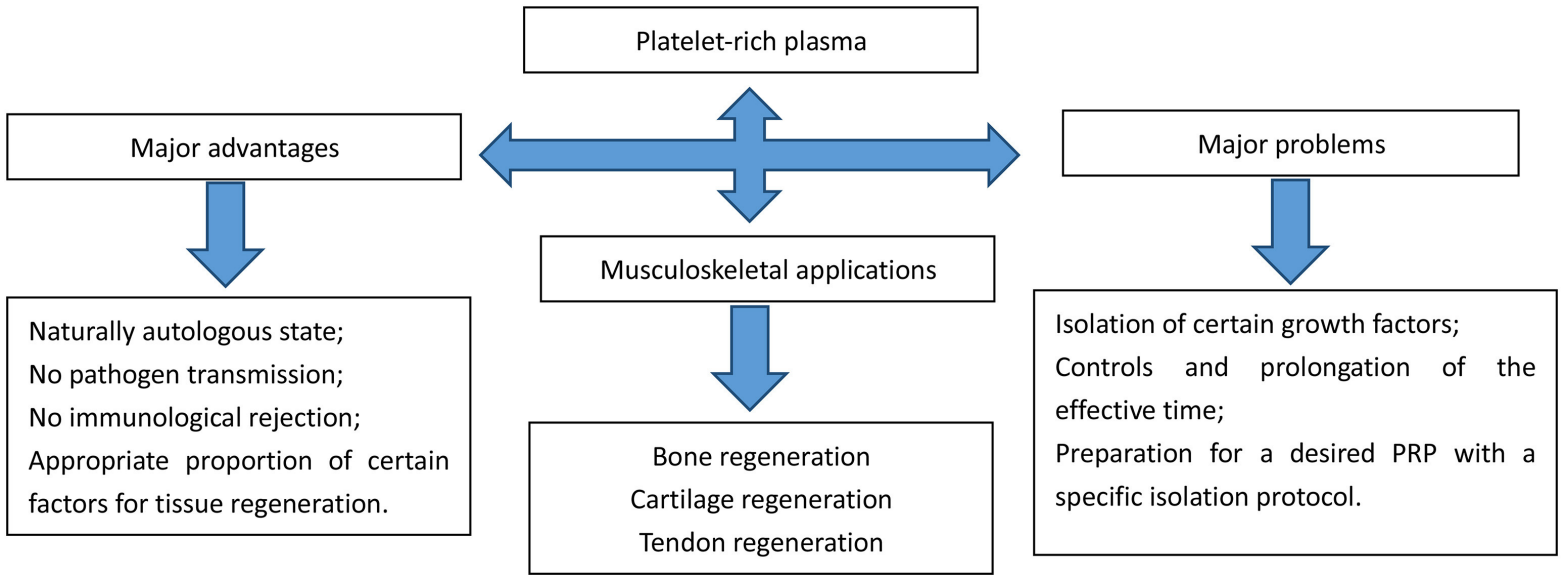
Advantages, applications, and problems of platelet-rich plasma.

In addition to stem cell therapy, blood derived products such as autologous conditioned serum and the latest orthopedic panacea, platelet rich plasma (PRP) has also been widely researched on its plentiful applications in various fields. Bone defects in the oral cavity and maxillofacial region are the major fields (Mehta, 2010); Soon later its applications extended across many fields including periodontal (Moghe et al., 2012; Morikuni et al., 2013) and oral and maxillofacial surgery (Albanese et al., 2013; Daif, 2013), aesthetic plastic surgery (Cervelli et al., 2009), spinal surgery (Okamoto et al., 2012), cardiac bypass surgery (Gravlee, 1994) and treatment of soft-tissue wounds or ulcers (Akhundov et al., 2012; Jiritano et al., 2013). The early introduction of PRP into clinical practice was suggested by Ferrari et al. (1987). PRP is an autologous platelet concentrate obtained from fresh whole blood by centrifugation. To be applied at the surgical site, PRP must be activated to induce platelet degranulation and fibrin polymerization, thus obtaining a clot usually called platelet gel (PG). PRP is naturally heterogeneous for various factors that exist in PRP preparation protocols including: (1) the initial quantity of platelets, (2) the applications of anticoagulants, (3) the use of leukocytes, and (4) the inclusions of activators resulting in different biological outcomes. Apart from naturally autologous state, and no risk of pathogen transmission or immunological rejection, PRP contains the appropriate proportion of certain factors necessary for wound healings (Marques et al., 2015) (referred to in Figure 1).

The biological characteristics of PRP rely on the concentration in platelets. Proper preparations can help PRP secrete many growth factors (GFs) at high concentrations, including transforming growth factor-β, platelet-derived growth factor, insulin-like growth factor, vascular endothelial growth factor and epidermal growth factor. Therefore, standardization for PRP is very important. But variation in PRP concentration usually leads to decreased and unstable repairing effect in tissue regeneration. There are a few dozens of protocols and purification systems for PRP isolation in the world. Different methods result in different PRP contents and concentrations as well as variant clinical outcomes. Most researches indicated that five times content of normal platelets can contribute to effective regeneration by PRP. And higher concentration does not induce a better result (Marx, 2004). The best concentration of PRP has not been decided yet. But different purification facilities and methods will result in different biological characteristics and variance in clinical outcomes (Yu et al., 2011). Kreuz and colleagues used different isolation kits and centrifugation for PRP preparation. The results turned out that Dr.PRP Kit could most effectively promote mesenchymal progenitor cell proliferation, compared with other methods. Moreover, some protocols could even exhibit a higher chondrogenic differentiation potential than others (Kreuz et al., 2015). Masoudi and colleagues also mentioned different modes of PRP preparation affected other aspects, like extended viability under low temperature and increased platelet yields with longer centrifugation period (Masoudi et al., 2016). Scientists have expected to combine PRP and stem cell in the field of tissue regeneration, especially bone, cartilage and tendon repair for a long time. However, the relationship between PRP preparation and stem cell activity is very controversial. There are a few important applications of PRP and stem cells in musculoskeletal regeneration in recent years. Rubio-Azpeitia and colleagues used platelet-rich plasma (PRP) three dimensional printing scaffold to induce mesenchymal stem cells differentiation, and found increased expression of collagen 1 and 3 in this model. Besides, other articular genes were upregulated as well in mesenchymal stem cells stimulated by PRP (Rubio-Azpeitia et al., 2017). Tang and colleagues experimented on rabbit derived PRP for chondrogenic induction of adiposed stem cells. Collagen II was significantly increased accompanied by aggrecan expression (Tang et al., 2015). However, Huber and colleagues discovered thrombin was effective in helping production of PRP, with an easier and faster style in comparison with other activation and purification method. However, they tested GFs like PDGF-AA, VEGF and VEGF, and found this kind of isolation method had no positive effects in promoting GFs secretion (Cares et al., 2016). PRP is vital for advancing proliferation of bone precursor cells in different animals, including rabbits and goats (Lin et al., 2013; Kim et al., 2014). In spite of rich potentials of direct applications of PRP for wound healings, combining PRP with bone marrow mesenchymal stem cells (BMSCs) for tissue regeneration applications becomes a rising interest for a long time. PRP is a significant supplement for PRP-gel delivery carrier for cell transplantation. In addition, it is mentioned that PRP can contribute to the proliferation of BMSCs and their differentiation into osteoblasts (Montgomery et al., 2011). The GFs in PRP have many functions, including accelerating angiogenesis and promoting collagen formation, thus leading to wound healing and tissue formation. Hence, PRP is thought to be an ideal origin of GFs for many applications.

According to the aspects discussed above, GFs originate from PRP and cooperate with stem cells in bone healing and soft tissue repair. This review is mainly aimed at the interaction of PRP derived growth factors with stem cells in various differentiation into mature somatic cells in musculoskeletal regeneration.

## Growth factors

Growth factors derived from PRP can contribute to tissue regeneration, by assisting cell migration, proliferation, differentiation and extra-cellular matrix synthesis (Gulotta et al., 2011; Isaac et al., 2012). However, the varying GF concentrations may have different biologic effects, resulting in the fact that individual differences in GF levels should be considered for reliable interpretation of the biologic functions and standardized application of PRP. Numerous GFs, including the basic fibroblast growth factor (bFGF), platelet-derived growth factor (PDGF), and transforming growth factor-β (TGF-β), which are released from PRP, can be detected during the early phase of wound healing (referred to in Table 1). Many of these GFs have a unique temporal expression profile and are thought to play an important role in the musculoskeletal regeneration. Generally, GFs are delivered locally to the bone and soft tissue regeneration site with tissue-engineered scaffolds, covered sutures, or dissolved in a fibrin-like encapsulation (Losi et al., 2013). Successful strategies to biologically augment tissue repair will require appropriate combination of GFs, responding host cells to the growth factor signaling and an optimal delivery method (Gulotta et al., 2011).

**Table 1:** Platelet-rich plasma derived Growth factors and their applications in musculoskeletal regeneration.

**Growth factors**	**tissue**	**Stem cells**	**species**	**functions**	**References**
bFGF	bone bone	BMSC BMSC	human rat	to induce calcium deposition; to support BMSCs expansion; to indicate severe bone lesion; to stimulate BMSCs differentiation into bone	Dines et al., 2007; Clendenen et al., 2010; Kawaguchi et al., 2010; Hata et al., 2013; Bai et al., 2014; Cheng et al., 2014
IGF	muscle bone	/ PDLSC	human human	to stimulate skeletal muscle regeneration to stimulate proliferation and osteogenic differentiation	Creaney and Hamilton, 2008; Kleplová et al., 2014; Martínez et al., 2016
TGF-β	bone bone cartilage bone	ADSC ADSC BMSC BMSC	human rabbit human dog	to stimulate bone deposition for successful fracture healing; osteogenic differentiation of ADSC; to stimulate chondrogenesis of BMSC; to stimulate new bone formation and angiogenesis.	Yamada et al., 2004; Zurita et al., 2010; Wang et al., 2012; Busilacchi et al., 2013; Elder and Thomason, 2014
VEGF	bone bone bone	BMSC BMSC BMSC	mouse/rabbit rat rat	to stimulate both angiogenesis, vasculogenesis and bone regeneration; To increase blood flow	Kim et al., 2010; El Backly et al., 2013; Bai et al., 2014
PDGF	bone bone bone tendon	BMSC BMSC PDLSC /	rat dog human human	to promote blood vessel and bone healing; to stimulate new bone formation and angiogenesis. to stimulate proliferation and osteogenic differentiation; to enhance tenocyte proliferation and promote synthesis of ECM	Yamada et al., 2004; Hu et al., 2009; Scheibel et al., 2011; Kleplová et al., 2014; Martínez et al., 2016.

*bFGF, basic Fibroblast Growth Factor; IGF, Insulin-Like Growth Factor; TGF-β, Transforming Growth Factorβ; VEGF, Vascular Endothelial Growth Factor; PDGF, Platelet Derived Growth Factor; MSCs, muscle derived stem cells; BMSCs, bone marrow stem cells; ADSCs, adipose derived stem cells; PDLSCs, periodontal ligament stem cells*.

### Basic fibroblast growth factor (bFGF)

PRP derived basic fibroblast growth factor (bFGF) is actively involved in osteogenesis and angiogenesis. Recombinant bFGF has been used for supporting BMSCs expansion, and it is able to induce an abundant calcium deposition (Cheng et al., 2014). Preclinical researches suggest that bFGF may be useful to promote bone healing (Kawaguchi et al., 2010; Hata et al., 2013). The use of recombinant bFGF after nailing of tibial fractures significantly improves fracture healing (Granchi et al., 2013). Recently, it has been demonstrated that bFGF circulating level predicts the outcome of a severe bone lesion (Clendenen et al., 2010). Besides, polymorphonuclear neutrophils (PMN) from healthy individuals' express different levels of FGF receptors in their cytosol (FGFR-1 and FGFR-4) and cytoplasmic membrane (FGFR-2) (Chamberlain et al., 2011). These receptors may capture bFGF, therefore explaining the inverse relation between bFGF and PMN number in leukocyte platelet rich plasma (L-PRP) samples at all-time points. In addition, PRP concentrates contain lymphocytes and sustain the production of new bFGF after the initial release. The effect of leukocytes is still controversial and can be either positive or negative, depending on tissue and underlying disease. Some authors have demonstrated that the presence of PMN can result in proinflammatory cell signaling and local tissue catabolism, whereas others have shown that macrophages are essential for debridement of damaged ligamentous tissue and for cytokine release mediating the repair process (Dines et al., 2007).

### Insulin-like growth factor 1 (IGF-1)

IGF-1 is able to stimulate proliferation and migration of fibroblasts and other local cells, and serves as a crosslink between muscle and bone with certain physical and biochemical cues (Bikle et al., 2015). Kabiri et al. reported that the PRP has rich concentration of a few GFs like IGF-1 TGF-βand bFGF, they also discussed its potential role in chondrogenic regeneration, especially in promoting chondrocyte proliferation and attachment as well as MSC differentiation. However, the direct role of PRP derived IGF-1 in bone and muscle regeneration is not illustrated (Kabiri et al., 2014). Creaney reported that PRP derived mechano growth factor (MGF), an isoform of IGF-1, could stimulate myoblast proliferation and it might have a potential role in improving musculoskeletal restoration. However, the short half-life limited its systemic applications. Therefore, MGF was only effective for regional use. Besides, the concentration of IGF-1 isoforms in PRP cannot sufficiently produce a promoting effect in muscle regrowth, which requires large quantification of raw material in bone and muscle repair (Creaney and Hamilton, 2008).

### TGF-β

TGF-β, one of the most abundant GFs released from PRP, is demonstrated to promote tendon healing in the absence of scar formation. Therefore, it is considered as a good candidate to reproduce a tendon-bone insertion site. Studies about the delivery of TGF-βat bone injury site are controversial. It was reported that high expression of TGF-β could lead to pathologic bone formation and collagen secretion. Therefore, it resulted in abnormal fibrotic synthesis and failure of ideal bone repair (Shehata et al., 2004). Busilacchi et al. found that PRP could be regulated by synthetic materials and secrete various GFs for adipose derived stem cell differentiation. Among them, TGF-β was obviously elevated and could stimulate bone deposition for successful fracture healing (Busilacchi et al., 2013).

### Vascular endothelial growth factor (VEGF)

VEGFs are a family of signaling proteins that function to stimulate both angiogenesis and vasculogenesis through a tyrosine kinase receptor–mediated signaling cascade (Kang et al., 2012). Several studies have examined VEGF augmentation of bone repairs, in different stages of bone healing, including inflammation, endochondral ossification, intramembranous ossification (Hu and Olsen, 2016). El Backly and colleagues designed a PRP based scaffold in combination with mesenchymal stem cells and they found VEGF secretion from PRP contributed to migration of endothelial cells, thus leading to angiogenesis and osteogenesis (El Backly et al., 2013). Kim used PRP for rat cranial bone defect model and discovered blood flow increase accompanied by upregulated VEGF expression *in vivo* (Kim et al., 2010).

### Platelet derived growth factor (PDGF)

Platelet-derived growth factor–BB (PDGF-BB) has been shown to improve healing in bone repair models. Recombinant PDGF can reduce osteogenic differentiation of mesenchymal cells *in vitro*. Hee et al. (2011) implanted a recombinant human PDGF-BB (rhPDGF-BB) together with a type I bovine collagen matrix, in order to improve the biomechanical function and morphologic appearance of the rotator cuff repair in an ovine model. 2 weeks results showed an augmentation in biomechanical strength and anatomic appearance. Platelet derived growth factors (PDGF), could be used after a rotator cuff tear, to induce proliferation and synthesis of tenocytes with appropriate ECM proteins(Scheibel et al., 2011). In a tendon injury, the supraspinatus tenocytes are not able to synthesize normal fibrocartilaginous extracellular matrix (ECM) or collagen II, but only collagens I and III (Venneri et al., 2007). In an *in vitro* study, PDGFs seemed to enhance tenocyte proliferation and promote synthesis of ECM (Scheibel et al., 2011). Hu and colleagues found that PRP could upregulate VEGF and PDGF expression and stimulate BMSCs differentiation into bone (Hu et al., 2009).

## Interaction of PRP derived growth factors with stem cells

### Growth factors and adipose-derived stem cells (ADSCs) in musculoskeletal regeneration

The use of adipose-derived stem cells (ADSCs) in bone regeneration requires a sound knowledge of optimized cell isolation and handling procedures as well as culture conditions because these can significantly affect their proliferative capacity, differentiation potential and gene expression. ADSCs express surface markers likeCD44, CD73, CD90, and CD105, but are negative for CD14, CD34, and CD45 (Gaiba et al., 2012; Khan et al., 2012).

There are a few *in vitro* studies about the potential effects of PRP released GFs on stem cell and their cooperation in tissue regrowth. Wang et al. discovered that PRP could efficiently improve proliferation and differentiation of adipose derived stem cells with secretion of TGF-1 and PDGF-AB. And the effect of PRP was more long-lasting than platelet-rich fibrin (Wang et al., 2012).

Besides, the ADSCs could also be strongly induced into chondrogenic differentiation by 20% PRP derived growth factors in a cartilage alike microenvironment (Shen et al., 2015).

The combined influence of PRP derived growth factors and ADSCs was also discussed for *in vivo* research. Van Pham et al. noticed that activation of PRP could significantly contribute to a certain differentiation of ADSCs and improve articular cartilage regeneration *in vivo* (Van Pham et al., 2013).

### Growth factors and bone marrow derived stem cells (BMSCs) in musculoskeletal regeneration

Mesenchymal stromal cells (MSCs) derived from a variety of sources possess the potential to be used in cell-based therapies. However, due to low proportion of primary MSCs present in bone marrowplastic adherence and *in vitro* expansion are essentialto proliferateBMSCs prior to clinical application.

There are a couple of important *in vitro* studies in this field. With the help ofchondroinductive TGF-β3, PRP and alginate contributed equally to chondrogenic development with their own characteristics. TGF-β3 was not a necessary factor for chondroinduction of BMSCs, but this processcould be hugely accelerated at the presence of TGF-β3 (Zurita et al., 2010). The alginate bead culture system is indicated to greatly improve successful chondroinduction of hBMSCs *in vitro*, and was therefore used to compare with PRP. Those cells which do not interact with alginate molecules remain rounded shapes until a pericellular matrix is created for connections (Elder and Thomason, 2014). This spherica shape contributes todifferentiation into chondrogenic tissues. In addition, integrin dependent cell-ECM reaction stimulated by PRP can greatly improve chondrogenic differentiation stimulated by TGF-β3. Besides, the expression of Col-2, Sox-9, and AGC could be upregulated at the presence of PRP and low TGF-β3 expression to stimulate BMSC chondrogenic differentiation, which was possibly affected by joint efforts of various GFs from PRP in cartilage regeneration (Elder and Thomason, 2014).

In addition, the combined effect was also evaluated in some *in vivo* studies. The PRP of 2-5% concentration could better increase BMSC osteogenic differentiation. In this process, the expression of PDGF and TGF-β released from PRP were greated increased, which contributed to a efficient and successful bone repair (Yamada et al., 2004). Bai et al. discovered that a joint application of VEGF and bFGF in BMSCs could significantly stimulate the osteogenesis and increased bone regeneration in a rat model. The addition of GFs could further contribute to the differentiation at the beginning of proliferation (Bai et al., 2014).

### Growth factors and periodontal ligament stem cells (PDLSCs) in musculoskeletal regeneration

The research on human periodontal ligament stem cells has also attracted the attention of many scientists. It is very easy to isolate the PDLSCs for its rich content from the teeth (Yeasmin et al., 2014). Moreover, the extraction and purification procedures are commonly adopted, which leads to no major harm to patients (Xiong et al., 2016).

Previous researches have reported potential impacts of PDLSCs on combination with PRP in bone regeneration *in vitro*, with increased ALP activity and osteogenic differentiation. Some scientists found that the ALP activity of PDLSCs increased following treatment with umbilical cord blood-platelet rich plasma (UCB-PRP) in a dose dependent manner up to a concentration of 2%. ALP activity decreased with higher concentration of UCB-PRP. The effects of UCB-PRP on calcium deposition were similar to those on proliferation and ALP activity (Lee et al., 2011). Treatment with 2% UCB-PRP resulted in the highest calcium depositions in HPDLSCs. The concentrations of platelet-derived growth factor-AB and transforming growth factor-β1 in UCB-PRP were investigated and found to be comparable to the amounts in peripheral blood. Besides, platelet derived growth factors isoforms (PDGF-AA, PDGF-BB, PDGF-AB), Insulin-like Growth Factor Binding Proteins 2 and 6 (IGFBP-2, IGFBP-6) were also closely involved in the process. Overall, UCB-PRP had beneficial effects on the proliferation and osteogenic differentiation of human periodontal ligament stem cells (Kleplová et al., 2014). Xu and colleagues also discovered that a significant enhancement of cell differentiation in a combined use of PDLSCs and PRP. Various GFs released from PRP contributed to improved cell sheet reconstruction, elevated osteogenic gene expression, including ALP, Runx2, Col-1, and OCN. All of these results displayed a stronger periodontal tissue regenerative capacity (Xu et al., 2014).

Martinez also found similar results from *in vivo* research. They used PRP and platelet-poor plasma in bone defect model. Increased expression of PDGF isoforms, including PDGF-AA,-BB, and AB as well as IGF isoforms, from both platelet concentrates could stimulate PDLSCs osteogenic stimulation and calcium deposition at a relatively low concentration (Martínez et al., 2016).

## Conclusion

Stem cells have become more and more important in regenerative medicine and can be isolated easily and reproducibly from human tissues. They have many advantages such as easy and long-term proliferation, multi-lineage potential and tolerance toward hypoxic environments. The application of stem cells from various tissues in diverse diseases and anti-aging treatments is very promising in regenerative medicine. PRP is most often considered as a bio-aggregate of GFs and used to supplement biomaterials. It can also be prepared with fibrinogen, which can be easily activated to form fibrin gel with thrombin or calcium. Soon later its applications extended across many fields including periodontal and oral and maxillofacial surgery, treatment of soft-tissue wounds and so forth. Further investigation will be focused mainly on the exact signaling pathways in the interaction between genetic molecules mediated or released from GFs derived from PRP and their effects in modification, migration, and differentiation of stem cells. Primary questions lie in: (1) how to isolate certain GFs that influence a specific stem cell. We know that PRP derived growth factors can induce stem cell differentiation, proliferation and adhesion. However, a few GFs are upregulated at the same time. Therefore, it is hard to confirm the exact effecting growth factor in the process. (2) how to control and prolong the effective time of each growth factor for the best use. Normally, GFs are active under certain conditions for a short period of time. Proteins can be inactivated by many physical and chemical cues. It brings some difficulty to clinical scenario applications. (3) how to successfully prepare a desired PRP with a specific isolation protocol. There are many isolation methods with different facilities under varies temperature, humidity, and blood origin. It is hard to reach a consensus for the best PRP purification protocol. More emphasis will be laid on this aspect in preclinical and clinical researches. Once we can elucidate the underlying mechanism completely, we will illuminate the road ahead and create more miracles in musculoskeletal regeneration.

## Author contributions

YQ, QH, WC, JS, XZ, YO, CF, and WY participated in its design, searched databases, extracted and assessed studies and helped to draft the manuscript. WY conceived the initial idea and the conceptualization, participated in the data extraction and analysis, and revised the manuscript. All authors read and approved the final manuscript.

### Conflict of interest statement

The authors declare that the research was conducted in the absence of any commercial or financial relationships that could be construed as a potential conflict of interest.
